# Genetic Characterization of Fungi Isolated from the Environmental Swabs collected from a Compounding Center Known to Cause Multistate Meningitis Outbreak in United States Using ITS Sequencing

**DOI:** 10.3390/pathogens3030732

**Published:** 2014-08-22

**Authors:** Irshad M. Sulaiman, Emily Jacobs, Steven Simpson, Khalil Kerdahi

**Affiliations:** Southeast Regional Laboratory, U.S. Food and Drug Administration, 60, Eighth Street NE, Atlanta, GA 30309, USA; E-Mails: emily.jacobs@fda.hhs.gov (E.J.); steven.simpson@fda.hhs.gov (S.S.); khalil.kerdahi@fda.hhs.gov (K.K.)

**Keywords:** genetic marker, fungal meningitis, microbiological indicators, rapid detection

## Abstract

A multistate fungal meningitis outbreak started in September of 2012 which spread in 20 states of the United States. The outbreak has been fatal so far, and has affected 751 individuals with 64 deaths among those who received contaminated spinal injections manufactured by a Compounding Center located in Massachusetts. In a preliminary study, Food and Drug Administration (FDA) investigated the outbreak in collaboration with Centers for Disease Control and Prevention (CDC), state and local health departments, and identified four fungal and several bacterial contaminations in the recalled unopened injection vials. This follow-up study was carried out to assess DNA sequencing of the ITS1 region of rRNA gene for rapid identification of fungal pathogens during public health outbreak investigations. A total of 26 environmental swabs were collected from several locations at the manufacturing premises of the Compounding Center known to have caused the outbreak. The swab samples were initially examined by conventional microbiologic protocols and a wide range of fungal species were recovered. Species-identification of these microorganisms was accomplished by nucleotide sequencing of ITS1 region of rRNA gene. Analysis of data confirmed 14 additional fungal species in the swabs analyzed.

## 1. Introduction

The primary mission of the U.S. Food and Drug Administration (FDA) is to enforce the Food, Drug and Cosmetic Act and regulate food, drug and cosmetic products. To evaluate adulteration in these products, FDA uses the presence of fungi in manufacturing and distribution areas as one of the regulatory action criteria and also to ensure that the firm is following good manufacturing practices (GMP). One way to achieve the said goal is establishing an environmental monitoring program, and verifying the effectiveness of pathogen control in the production as well as distribution areas where the food, drug or cosmetics are exposed to potential contaminants. The environmental monitoring program provides significant information on the quality of the aseptic processing environment when a given batch of food, drug or cosmetic is being manufactured together with environmental trends of the manufacturing area. A proper monitoring program also identifies potential routes of contamination, allowing for implementation of corrections before product contamination occurs [1].

A multistate fungal meningitis outbreak started in mid-September of 2012. This outbreak affected individuals who had received contaminated injections (that included betamethasone, cardioplegia, and triamcinolone solutions), manufactured and distributed by a Compounding Center located in Massachusetts to outpatient facilities in 20 states of the United States. The firm voluntarily terminated all operations, surrendered its license, and announced a recall of all their products on October 3, 2012. In the beginning, FDA investigated this outbreak in collaboration with U.S. Centers for Disease Control and Prevention (CDC), state and local health departments, and analyzed the unopened vials of spinal injections. Four fungal (including *Aspergillus tubingensis*, *Aspergillus fumigatus*, *Cladosporium* sp., and *Penicillium* sp.) and several bacterial contaminations (*Bacillus circulans*, *Bacillus firmus*, *Bacillus flexus*, *Bacillus halmapalus*/*horikoshii*, *Bacillus idriensis*, *Bacillus lentus*, *Bacillus niabensis*, *Bacillus niacini*, *Bacillus nealsonii*, *Bacillus pumilus*, *Bacillus simplex*, *Bacillus subtilis* group, *Brevibacillus choshinensis*, *Kocuria rosea*, *Lysinibacillus* sp., *Paenibacillus barengoltzii*/*timonensis*, *Paenibacillus pabuli*/*amolyticus*,) were identified in the recalled unopened vials shipped by this Compounding Center [2,3,4]. Later, while analyzing the tissue and human fluids of patients infected with above contaminated injections CDC confirmed *Exserohilum rostratum* as the predominant fungal infection in this outbreak, and identified 22 additional fungal species (including *Aspergillus* sp., *Cladosporium* sp., *Alternaria* sp., *Bipolaris* sp., *Chaetomium* sp., coelomycete fungus, *Epicoccum nigrum*, Paecilomyces, *Penicillium* sp., *Scopulariopsis brevicaulis*, and *Stachybotrys chartarum*). Furthermore, CDC was able to isolate four more fungal species (*Cladosporium cladosporioides*, *Exserohilum rostratum*, *Rhodotorula laryngis* and *Rhizopus stolonifer*) from some of the unopened vials examined. The outbreak was deadly and affected a total of 751 individuals resulting in 64 deaths, as of October 23, 2013 [4].

To date, the taxonomy of fungi is in a constant state of flux. The kingdom fungi have been estimated to be approximately 1.5–5 million species that grow as hyphae, with about 5% of these having been formally classified. The 200,000 known fungal species make up a quarter of the biomass of the earth. Of these, approximately 100,000 genera have been identified as the mold species, and 80 of them are known to cause illness in humans. In recent years, DNA based molecular characterizations of fungi have been extremely successful to understand the extents of genetic diversity at inter- and intra- species level. Phylogenetic studies have further revealed a monophyletic origin of fungi with a closer relationship to animals than plants. Of various genetic marker characterized, the internal transcribed spacer (ITS) region of rRNA gene has been most widely sequenced in fungi [5,6,7]. More recently, the ITS region was also proposed as the universal DNA barcode marker for fungal taxonomy and species identification [8].

This follow-up study was conducted to determine a possible cause of the multistate fungal meningitis outbreak. To achieve the goal, 26 environmental swab samples were collected from various locations of the manufacturing premises of the compounding company following the guidelines of FDA. These swabs were examined for the presence of fungal species initially by conventional microbiologic protocols. Afterwards, the recovered fungal isolates were genetically identified by performing DNA sequencing of PCR-amplified ITS1 region of rRNA gene. Species-specific variation was obtained based on ITS1 sequence characterization. Results of study clearly indicated the ITS1 region to be a consistent genetic marker for rapid detection and differentiation of human-pathogenic fungi, considered as microbiological indicators in the environment of public health importance.

## 2. Results and Discussion

### 2.1. Recovery of Fungal Isolates from Environmental Swab Samples using Selective Enrichment Media

Of the 26 swab samples examined, fungal isolates were recovered from 25 of the swabs from both liquid (Sabouraud Dextrose Broth) and semi-solid (Malt Extract Agar) selective media. These media are widely used for the determination of fungistatic activity and cultivation of pathogenic fungi from food, pharmaceutical and cosmetic products (sterile and non-sterile) and in performing Environmental Monitoring and Sterility Testing [9,10].

### 2.2. Genetic Diversity of Recovered Fungal Species from Environmental Swabs at the ITS Locus

Genomic DNA was extracted and used to amplify the ITS1 region of rRNA gene, for all of the 25 recovered fungal isolates from the environmental swab samples. Nucleotide sequence of ITS1 region of rRNA gene was obtained for all 25 recovered isolates by performing bi-directional sequencing of the PCR amplified fragments. DNA sequences from both forward and reverse primers were assembled, manually edited and compared with the known sequences available in the GenBank using BLAST algorithm. The PCR was repeated and the resultant PCR products were sequenced again when the sequence data was obtained with low quality score. The DNA sequences were considered identical if they shared 100% identity with the known ITS1 sequences available in GenBank. A species-specific genetic variation was observed in the ITS1 region of rRNA gene characterized. However, the intra-specific genetic variation was not noticed at this locus.

Multiple alignments of the generated ITS1 region of rRNA nucleotide sequences confirmed the presence of 14 distinct fungal species belonging to 12 different genus that included *Alternaria alternata*, *Alternaria tenuissima*, *Aspergillus niger*, *Aureobasidium pullans*, *Aureobasidium proteae*, *Chaetomium globosum*, *Cryptococcus saitoi*, *Filobasidium capsuligenum*, *Leptosphaerulina chartarum*, *Mucor racemosus*, *Mucor velutinosus*, *Penicillium chrysogenum*, *Rhizomucor variabilis*, *Rhizopus oryzae*, and *Rhodotorula glutinis* (Table 1).

**Table 1:** Species-specific identification of fungal isolates recovered from environmental swabs collected from the Compounding Company premises by sequence characterization of ITS1 region of rRNA gene.

Species Identified	Swab Sample Analyzed	Sequence Type	% Similarity (bp)	*Reference
*Alternaria alternata*	ES-24	rRNA	100 (246)	KC568287, this study
*Alternaria tenuissima*	ES-4	rRNA	100 (211)	JX867219, this study
*Aspergillus niger*	ES-8	rRNA	100 (268)	KC119204, this study
*Aureobasidium proteae*	ES-13	rRNA	100 (229)	JN712490, this study
*Aureobasidium pullulans*	ES-22	rRNA	100 (221)	AY139394, this study
*Chaetomium globosum*	ES-9	rRNA	100 (210)	JX406510, this study
*Chaetomium globosum*	ES-18	rRNA	100 (256)	AY429056, this study
*Chaetomium globosum*	ES-19	rRNA	100 (256)	AY429056, this study
*Chaetomium globosum*	ES-20	rRNA	100 (256)	AY429056, this study
*Cryptococcus saitoi*	ES-25	rRNA	100 (245)	KC254024, this study
*Filobasidium capsuligenum*	ES-16	rRNA	100 (214)	EF532832, this study
*Leptosphaerulina chartarum*	ES-5	rRNA	100 (299)	JX442978, this study
*Leptosphaerulina chartarum*	ES-21	rRNA	100 (291)	EU272493, this study
*Mucor racemosus*	ES-10	rRNA	100 (290)	AY213660, this study
*Mucor racemosus*	ES-11	rRNA	100 (290)	AY213660, this study
*Mucor racemosus*	ES-12	rRNA	100 (290)	AY213660, this study
*Mucor racemosus*	ES-23	rRNA	99 (289)	AY213660, this study
*Mucor velutinosus*	ES-1	rRNA	100 (279)	JX120704, this study
*Mucor velutinosus*	ES-2	rRNA	100 (279)	JX120704, this study
*Mucor velutinosus*	ES-3	rRNA	100 (279)	JX120704, this study
*Penicillium chrysogenum*	ES-6	rRNA	100 (211)	JF807949, this study
*Penicillium chrysogenum*	ES-7	rRNA	100 (211)	JF807949, this study
*Rhizomucor variabilis*	ES-17	rRNA	100 (253)	JF904893, this study
*Rhizopus oryzae*	ES-14	rRNA	100 (239)	JN227043, this study
*Rhodotorula glutinis*	ES-26	rRNA	100 (220)	JQ993380, this study

*Representative sequences generated in this study have been deposited in GenBank, with accession numbers KC852046 to KC8522066, and KF445073 to KF445076.

The engendered DNA sequences belonging to ITS1 region of rRNA gene of all the isolated fungal species except one isolate matched 100% with the published sequences available in the GenBank. The recovered isolate MOSS23 (*M. racemosus*) displayed single nucleotide mutation at position 153 (“G” to “A”) as compared to several existing DNA sequences in the public domain of this species (GenBank accession no. JF440624, GenBank accession no. AY213660).

### 2.3. DNA Sequencing of ITS region in Molecular Typing of Human-pathogenic Microorganisms

Molecular genetic characterization of microorganisms has been extensively exploited for rapid detection and differentiation of a human-pathogenic species from sporadic cases, surveillance and outbreak samples of public health importance. These tools have provided correct identification of responsible infectious species, and helped in conducting epidemiologic investigations to determine the transmission routes. The rRNA genes are the most abundant housekeeping genes present in all organisms often with several copies evolving at a slower evolutionary rate [11,12]. Thus, this locus has been extensively used as the genetic marker to understand the phylogenetic relationship, and in developing diagnostic assays for rapid detection and differentiation of pathogenic species and their vectors [13,14,15,16,17].

Fungi encompass the second largest kingdom of eukaryotes that include human-pathogenic yeast and molds as well. Therefore, finding a suitable genetic marker for fungal taxonomy has been a daunting task. Although a number of genes have been typed as a genetic marker, the ITS1 region of rRNA gene has been the most recognized locus for species identification and sub-generic phylogenetic relationship in fungi. However, the ITS2 region of rRNA region has also been exploited for above purposes [5,6,7,18,19,20,21,22,23]. In a recent comprehensive study, ITS locus is recommended as the universal DNA barcode marker for fungi [8]. The ITS1 sequence characterization of ITS1 regions has identified 40 species of human-pathogenic yeasts [6]. ITS based multiplex PCR assay identified five *Rhizopus* species including *Rhizopus oryzae* that causes meningitis in humans [21]. The ITS locus also revealed phylogenetically valid information for correct identification of 201 strains of clinically important molds belonging to 44 different species, while performing multilocus DNA sequence comparisons [24]. The ITS region of *Rhizopus* microspores was also utilized as a marker in an outbreak investigation of intestinal mucormycosis infection [25]. More recently, the ITS region (~350 bp) was also exploited for performing the targeted PCR and DNA sequencing in the detection of fungal DNA in clinical samples (human body fluids and tissues) during the investigation of this multistate outbreak of fungal meningitis and other infections. Using this genetic marker, *Exserohilum rostratum* DNA was detected and confirmed in 123 samples from the 114 case-patients, and *Cladosporium* sp. DNA in one sample from one case-patient [4].

### 2.4. Public Health Significance of the Presence of Fungi in Environment

In this study, we discovered fungi in 96% of the 26 environmental swab samples examined from the premises of the Compounding Company located in Massachusetts, and performed sequencing for these recovered isolates at the ITS1 region of rRNA locus. Analysis of data of the generated ITS1 region of rRNA gene sequences confirmed the presence of 14 distinct fungal species belonging to 12 different genera among the 26 swabs analyzed. Majority of the recovered fungal species from swabs (this study) have been reported to thrive in damp places (home or environment), and thus their presence is considered an indicator of moist and humid conditions. Several of the recovered fungal species such as *Chaetomium globosum* were reported to grow successively in water damaged buildings especially when they occur due to leakage through roofs and defective plumbing installations. In a study focusing on potential health effects of fungal exposure in the indoor environments, an extensive ITS sequencing was employed to characterize and compare the fungal flora isolated from the indoor dust collected from two nonindustrial buildings; one of the buildings was moisture damaged and the other was undamaged [22].

The presence of fungal species has been considered as environmental microbiological indicators [26]. Furthermore, some of the recovered fungi (this study) have been described to cause cutaneous and respiratory infections, and chronic fungal meningitis in humans that include several species belonging to *Alternaria*, *Aspergillus*, *Aureobasidium*, *Chaetomium*, *Mucor* and *Penicillium* genera [27,28,29,30,31,32]. In addition, fungi are known to initiate three major environmental conditions that include: (i) structural integrity of a building, (ii) negative aesthetic effects *i.e.* unpleasant to live in a building due to its look and foul odor, and (3) adverse health effects in sensitive individuals [33,34]. Fungi have also been used as biological indicators for measuring the indoor air quality of a building, and its impact on the health and well-being of inhabiting humans [35].

While comparing the results obtained from this study on characterization of the environmental swabs collected from the premises of the Compounding Company that was involved in manufacturing infected injections, it is important to mention that four fungal species were (*Aspergillus tubingensis*, *Aspergillus fumigatus*, *Cladosporium* species, and *Penicillium* species) isolated from the same lot of unopened vials of infected injections that caused the multistate fungal meningitis outbreak, in an earlier study carried out by FDA [4]. In addition, CDC identified 22 additional fungal species (*Aspergillus* sp., *Alternaria* sp., *Bipolaris* sp., *Chaetomium* sp., *Cladosporium* sp., coelomycete fungus, *Epicoccum* nigrum, *Exserohilum rostratum*, *Paecilomyces*, *Penicillium* sp., *Scopulariopsis brevicaulis*, and *Stachybotrys chartarum* with *Exserohilum rostratum* as the most prevalent species) in the infected patients, and four fungal species (*Cladosporium cladosporioides*, *Exserohilum rostratum*, *Rhodotorula laryngis* and *Rhizopus stolonifer*) in the unopened vials [4]. Of the 14 distinct fungal species (*Alternaria alternata*, *Alternaria tenuissima*, *Aspergillus niger*, *Aureobasidium pullans*, *Aureobasidium proteae*, *Chaetomium globosum*, *Cryptococcus saitoi*, *Filobasidium capsuligenum*, *Leptosphaerulina chartarum*, *Mucor racemosus*, *Mucor velutinosus*, *Penicillium chrysogenum*, *Rhizomucor variabilis*, *Rhizopus oryzae*, and *Rhodotorula glutinis*) recovered from the environmental swab samples in this study, some the fungal species were also recovered earlier in the unopened vials (such as *Aspergillus* sp., *Penicillium* sp. *Rhizopus* sp., and *Rhodotorula* sp.) as well as in the infected patients (*Aspergillus* sp., *Alternaria* sp., *Chaetomium* sp., and *Penicillium* sp.). *Exserohilum rostratum* was not recovered from the environmental swabs examined.

Recovering a large number of fungal species in the environmental swab samples analyzed in this study may be explained as the initial inspection carried out at the Compounding Company premises revealed that: (i) the firm’s environmental monitoring program yielded microbial isolates (bacteria and mold) within the clean room 1 and clean room 2 used for the production of sterile drug products between January 2012 and September 2012, and (ii) the firm was shutting off the air conditioning from 8:00 pm to 5:30 am in these clean rooms [4].

## 3. Experimental Section

### 3.1. Environmental Swab Sample Characterization and Recovery of Fungal Isolates

To recover fungi from the 26 environmental swabs, each swab was placed in a sterile plastic bag containing 100 mL of sterile Sabouraud Dextrose Broth (BD Difco^TM^, Sparks, MD, USA), and incubated at 25 °C for 5 days. After completion of 5 days of incubation, the broth was streaked to Malt Extract Agar (BD Difco^TM^) plates, and incubated at 25 °C for additional 7 days. These plates were read and growth was recorded after 24 h intervals every day for the next 7 days. Fungi observed on any of the agar plates were examined by staining and microscopy initially. Afterward, the recovered isolates were genetically characterized at molecular level for species identification. An unopened sterile swab was also tested as negative control. Furthermore, *Candida albicans* (ATCC #10231) and *Aspergillus brasilensis* (ATCC #16404) cultures were carried through the analysis to serve as positive culture controls. The swabbing was done on critical contact surfaces (with product and container) that included biological safety cabinet, equipment surfaces, and floors and walls of clean room of the manufacturing premises.

### 3.2. DNA Extraction

For genomic DNA extraction, a small portion (<100 mg) of fungal specimen was homogenized using a clean and autoclaved mortar and pestle, if needed liquid nitrogen was used. The homogenate was purified by RNeasy Plant Mini Kit DNA, following manufacturer’s protocol and recommendations for the purification of total genomic DNA from fungi (QIAGEN, Valencia, CA, USA). The concentration of purified DNA samples was measured at 260-nm absorbance using a NanoDrop-1000 spectrophotometer (NanoDrop Technology, Rockland, DE), and stored at –20 °C until used.

### 3.3. PCR Amplification

To amplify the fragments of ITS1 region of rRNA gene, a previously described forward (5'-TCC GTA GGT GAA CCT GCG G-3') and reverse (5'-GCT GCG TTC TTC ATC GAT GC-3') primer set [6,18,36] was used, with modifications in PCR conditions. For PCR, a total of 50 µL PCR reaction consisted of 25 µL of HotStarTaq Master Mix (QIAGEN), and 25 µL of a solution containing 200 nM of each primer, 1.5 mM of additional MgCl_2_ (Promega, Madison, WI, USA) and template DNA (50 ng) diluted in PCR grade water. The QIAGEN HotStarTaq Master Mix is a premixed solution containing HotStarTaq DNA Polymerase, PCR Buffer, and dNTPs with a final concentration of 1.5 mM MgCl_2_ and 200 µM each dNTP. The PCR reactions were run for 35 cycles (each cycle is 94 °C for 45 s, 55 °C for 45 s, and 72 °C for 60 s) in a GeneAmp PCR 9700 thermocycler (Applied Biosystems, Foster City, CA, USA), with an initial hot start (94 °C for 15 min) and a final extension (72 °C for 10 min). The PCR products were analyzed by agarose gel electrophoresis, and visualized after ethidium bromide staining.

### 3.4. DNA Sequencing

The amplified PCR products were enzymatically cleaned before cycle sequencing, 3 μL of ExoSAP‐IT (USB Corporation, Cleveland, OH, USA) was added to 5μL of each amplified PCR product. The mixture was incubated at 37 °C for 20 min followed by 80 °C for 15 min on a GeneAmp PCR 9700 thermocycler (Applied Biosystems). The purified PCR products were sequenced using AB Big-Dye 3.1 dye chemistry and AB 3500 XL automated DNA sequencers (Applied Biosystems) with sequencing reaction competed for 25 cycles (each cycle is 96 °C for 30 s, 50 °C for 15 s, and 60 °C for 4 min) and hold at 4 °C in a GeneAmp PCR 9700 thermocycler (Applied Biosystems). The cycle sequencing reactions contained 2 μL of cleaned PCR product, 1 μL of BigDye Terminator v3.1 Ready Reaction Mix, 2 μL of 5× Sequencing Buffer, 1.6 pmol of Forward or Reverse sequencing primer, and water in a final volume of 20 μL. Sequencing reactions were cleaned up with the Performa® DTR Gel Filtration Cartridges following manufacturer’s protocol (Edge Bio, Gaithersburg, MD, USA).

### 3.5. Data Analysis

The accuracy of nucleotide sequence was confirmed by performing two-directional sequencing, and by sequencing of a new PCR product if necessary. Multiple alignments of the nucleotide sequences were accomplished by BioEdit and Geneious programs with manual adjustments. The generated nucleotide sequences of ITS1 region of rRNA gene of recovered fungal species were deposited in GenBank database under accession number KC852046 to KC8522066, and KF445073 to KF445076.

## 4. Conclusions

Sequence characterization of the ITS1 locus can be used for the detection and differentiation of fungi from the environmental swab samples. This communication also reports for the first time the presence of 14 indicator fungal species isolated from the environmental swabs collected from the Compounding Center who previously manufactured the methylprednisolone injections contaminated with molds [2,3]. DNA sequencing of the ITS1 region of rRNA gene can be used for rapid identification of fungal pathogens during outbreak investigations of public health importance. 
